# An Overview of Mobile Colistin Resistance (mcr) Genes in Gram-Negative Bacilli

**DOI:** 10.7759/cureus.109203

**Published:** 2026-05-19

**Authors:** Manasi V Yadav, Satyajeet Pawar, Satish Patil

**Affiliations:** 1 Department of Microbiology, Krishna Institute of Medical Sciences, Krishna Vishwa Vidyapeeth (Deemed to be University), Karad, IND

**Keywords:** anti-microbial resistance, colistin resistance, mcr genes, mdr, molecular detection, plasmid-mediated resistance, pseudomonas aeruginosa

## Abstract

The increasing spread of mobile colistin resistance (*mcr*) genes is becoming a major concern in the treatment of infections caused by multidrug-resistant Gram-negative bacilli. Colistin is often used as a last treatment option, but the emergence of *mcr* genes is reducing its effectiveness. These genes are most commonly found in bacteria, such as *Escherichia coli*, *Klebsiella pneumoniae, *and* Salmonella, *and have also been reported, though less frequently, in organisms like *Pseudomonas aeruginosa*. This review provides an overview of the occurrence, diversity, and mechanisms of *mcr* genes in Gram-negative bacilli. These genes are usually carried on plasmids, which allows them to spread easily between different bacteria. They produce enzymes that modify lipid A in the bacterial outer membrane, reducing the ability of colistin to bind and act effectively. In addition, changes in chromosomal regulatory systems such as polymyxin resistance A and B *(pmrAB)*, phosphate regulon P and Q (*phoPQ), *and polymyxin adaptive resistance R and S (*parRS)* can further increase resistance. The spread of *mcr* genes is mainly driven by horizontal gene transfer, making it easier for resistance to move across different bacterial species and environments. From a clinical point of view, infections caused by *mcr*-positive bacteria can make treatment more difficult, increase the risk of complications, and put more pressure on healthcare systems. Therefore, early detection, regular monitoring, and careful use of antibiotics are important to control the spread of resistance. Understanding how these genes spread and persist in different environments will be important for developing better strategies to manage this growing problem.

## Introduction and background

Antimicrobial resistance is a growing global public health threat that compromises the effectiveness of life-saving antibiotics and increases the burden of infectious diseases worldwide. Recent global surveillance reports (2025) indicate that approximately one in six bacterial infections are resistant to commonly used antibiotics, highlighting the growing burden of multidrug-resistant (MDR) infections worldwide, particularly among Gram-negative bacteria in healthcare settings, and posing significant challenges to treatment and infection control [1].

In the current scenario, colistin has become one of the last lines of defense against life-threatening bacterial infections caused by MDR Gram-negative pathogens [2]. It is particularly important for the treatment of infections caused by Gram-negative bacilli, a major opportunistic pathogen frequently associated with hospital-acquired infections, especially in immunocompromised patients and those with chronic illnesses [3]. However, the growing problem of antimicrobial resistance has significantly worsened this situation. *Pseudomonas aeruginosa* is inherently difficult to treat due to its intrinsic resistance mechanisms and its ability to acquire resistance to multiple antibiotic classes. Increasing reports of colistin-resistant Gram-negative bacilli further limit available therapeutic options and pose a serious clinical challenge [4]. Until recently, resistance to colistin was thought to arise mainly from chromosomal mutations affecting lipid A modification pathways, meaning that resistance was not easily transferable between bacteria [5]. This understanding changed in 2015 with the discovery of plasmid-mediated mobile colistin resistance (*mcr*) genes, which can be transferred horizontally between bacteria via plasmids, facilitating the rapid spread of colistin resistance [6]. The 10 families of *mcr* genes (*mcr*-1 to *mcr*-10) have been identified worldwide. These genes have been predominantly reported in Enterobacteriaceae such as *Escherichia coli* and *Klebsiella pneumoniae* [7]. Although their occurrence in *P. aeruginosa* remains relatively rare, the detection of *mcr* genes in this species is of particular concern due to its persistence in hospital environments and its ability to cause severe infections in high-risk patients [8]. This review focuses on the emerging issue of colistin resistance in Gram-negative bacilli, particularly in Enterobacterales and *P. aeruginosa*, with emphasis on the appearance and potential dissemination of *mcr* genes. Understanding the role of these resistance determinants in clinical settings is essential for anticipating future treatment challenges and strengthening infection prevention and antimicrobial stewardship strategies.

## Review

Search strategy

This narrative scoping review evaluated studies on *mcr* genes in MDR Gram-negative bacilli and their associated resistance mechanisms. Literature published in English between January 2005 and December 2025 was retrieved from databases including PubMed, Scopus, Web of Science, Embase, and Science Direct. A structured search strategy using Boolean operators combined terms such as “colistin resistance,” “*mcr*,” and key Gram-negative pathogens (e.g., *P. aeruginosa*, *K. pneumoniae*, *E. coli*). Studies were included if they addressed the occurrence, mechanisms, or dissemination of *mcr* genes, while duplicates, reviews, and studies lacking relevant data were excluded. Study selection followed PRISMA guidelines, involving screening of titles, abstracts, and full texts. Study quality was assessed based on design, sample size, and methodological clarity. Due to heterogeneity among studies, findings were synthesized using a narrative qualitative approach without meta-analysis or statistical pooling. Reference lists were also screened to identify additional relevant studies.

Mechanisms of colistin resistance in *P. aeruginosa*

Plasmid-Mediated mcr Genes

A major emerging threat is the presence of *mcr* genes (*mcr*-1 to *mcr*-10), which encode phosphoethanolamine (PEtN) transferases. Similar to chromosomal PEtN enzymes, which add PEtN to lipid A and confer resistance. These genes, ranging from *mcr*-1 to *mcr*-10, encode PEtN transferases. These enzymes catalyze the transfer of PEtN to the phosphate groups of lipid A in the lipopolysaccharide (LPS) layer. This modification reduces the net negative charge of lipid A, thereby decreasing the electrostatic binding of colistin to the bacterial outer membrane and resulting in resistance [5]. These mobile colistin *mcr* genes are located on plasmids, allowing horizontal transfer between bacteria across species and environments, greatly amplifying their spread [9]. The first discovered *mcr*-1 was identified in *E. coli* isolates and has since been found in many Gram-negative bacilli, including *P. aeruginosa* in some reports, posing a global public health risk [10]. Although *mcr* genes are more widely reported in Enterobacteriaceae, sporadic presence of *mcr*-1 in colistin-resistant *P. aeruginosa* has been documented. These plasmid-borne determinants allow horizontal mobility of resistance [11].

Chromosomal Regulatory Systems (Two-Component Systems)

Chromosomal mutations in two-component regulatory systems play a central role in colistin resistance. Key systems include *pmrAB*, *phoPQ*, *parRS*, *colRS*, and *cprRS*. Mutations in these regulatory pathways lead to constitutive activation of lipid A modification mechanisms, resulting in reduced colistin binding and increased resistance [12,13].

Efflux Pump Overexpression

Increased expression of efflux pumps, like membrane fusion protein A, multidrug efflux transporter B, and outer membrane protein M (MexAB-OprM), can reduce the accumulation of colistin in bacterial cells, contributing to decreased susceptibility. Increased expression of efflux pumps is another important mechanism that contributes to reduced susceptibility to colistin. In *P. aeruginosa*, efflux systems, such as MexAB-OprM, act as active transport channels that pump antibiotics out of the bacterial cell. When these pumps are overproduced, they remove colistin more efficiently, preventing its accumulation inside the cell. This reduces the effective drug concentration and limits its interaction with the bacterial outer membrane, ultimately leading to decreased susceptibility [14].

Lipopolysaccharide Modification or Loss

Alterations in LPS structure represent a key resistance mechanism. Mutations in genes involved in lipid A biosynthesis (such as *lpxA*, *lpxC*, and *lpxD*) can lead to structural modification or complete loss of LPS. Colistin normally binds to lipid A through electrostatic interactions with negatively charged phosphate groups, displacing stabilizing divalent cations such as Ca²⁺ and Mg²⁺, which leads to membrane disruption and bacterial cell death. However, modification or loss of LPS reduces these binding sites, thereby diminishing colistin efficacy [15].

Biofilm Formation

Biofilm formation contributes to colistin tolerance by creating a protective matrix that limits antibiotic penetration. This enables bacterial persistence and survival even under antimicrobial pressure [16].

*mcr* genes: diversity and distribution

mcr Gene Families

*mcr* genes encode PEtN transferases that catalyze the addition of PEtN to lipid A, thereby reducing the net negative charge of LPS and decreasing colistin binding [2]. The first plasmid-mediated colistin resistance gene, *mcr*-1, was identified in *E. coli* from animals and humans in China in 2015, marking a paradigm shift in the epidemiology of colistin resistance [17]. The 10 major *mcr* gene families (*mcr*-1 to *mcr*-10) and numerous allelic variants have been described [6]. Although these genes share functional similarity, they differ in sequence homology, genetic context, plasmid incompatibility groups, and host range, suggesting multiple evolutionary origins [18]. Among them, *mcr*-1 is the most prevalent and widely disseminated, whereas *mcr*-2, *mcr*-4, *mcr*-5, and *mcr*-8 appear sporadically, and *mcr*-9 and *mcr*-10 are often associated with inducible or low-level resistance [19].

mcr Genes in P. aeruginosa

Plasmid-mediated *mcr* genes are predominantly reported in Enterobacterales; however, emerging evidence confirms their presence in non-fermenting Gram-negative bacilli, including *P. aeruginosa* [20]. Several studies have documented the detection of *mcr*-1 in clinical *P. aeruginosa* isolates, indicating that this opportunistic pathogen can acquire mcr determinants via horizontal gene transfer [21]. In rare cases of co-occurrence of multiple *mcr* genes (e.g., *mcr*-1 and *mcr*-3) within the same *P. aeruginosa* isolate have been reported, raising concerns about the accumulation of resistance mechanisms in already multidrug-resistant strains [22].

Global Spread of mcr Genes

Since their first identification, *mcr* genes have been reported worldwide across Asia, Europe, Africa, the Americas, and Oceania [15]. Among them, *mcr*-1 is the most widespread, having been detected in over 60 countries and across diverse ecological settings, including humans, livestock, companion animals, food products, wastewater, soil, and aquatic environments [23]. Other variants, ranging from *mcr*-2 to *mcr*-10, have also been identified globally in a variety of Gram-negative bacilli such as *Klebsiella*, *Salmonella*, *Enterobacter*, *Citrobacter*, *Aeromonas*, and *Acinetobacter* species [9].

mcr-1

*mcr*-1, first reported in 2015 in China from *E. coli* isolated from animals, food sources, and humans, remains the most widely distributed and clinically significant *mcr* variant. It has now been identified in more than 60 countries and is predominantly associated with *E. coli *and *K. pneumoniae*. Compared to other *mcr* variants, *mcr*-1 shows the strongest evidence for human clinical impact, largely due to its frequent localization on highly transmissible plasmids and its stable functional activity. While it is commonly detected in both human and animal isolates, its widespread occurrence across clinical settings distinguishes it from variants that are more restricted to environmental or livestock reservoirs. Although several amino acid substitutions (e.g., E246K and G213V) have been reported among *mcr*-1 variants, these generally have minimal impact on resistance levels, suggesting that the enzyme remains structurally stable and functionally conserved. This stability likely contributes to its global dissemination and persistence [24].

mcr-2

*mcr*-2 is mainly associated with livestock and is most commonly detected in *E. coli* isolates from pigs. Compared to *mcr*-1, its global distribution remains limited, with relatively few studies reporting its occurrence. This variant was first identified in 2016 in Belgium. It confers resistance to colistin through the same general mechanism of lipid A modification. *mcr*-2 shares approximately 80% amino acid identity with *mcr*-1 and retains similar catalytic residues responsible for its activity. The slight differences in membrane topology and a limited number of substitutions within the catalytic region have been observed. These variations may influence enzyme performance, resulting in slightly reduced efficiency and lower transmission potential compared to *mcr*-1. This could partly explain its more restricted dissemination and lower prevalence [25].

mcr-3

*mcr*-3 has been identified in multiple studies from both human and animal samples, particularly in Asian regions. It is found in a wider range of organisms, including *E. coli*, *Salmonella*, and *Aeromonas*, indicating a broader host range. It was reported in 2017 in China. Like other *mcr* variants, it confers resistance to colistin by modifying lipid A. *mcr*-3 is considered more genetically diverse than *mcr*-1 and *mcr*-2. It shows greater variation in its amino acid sequence, particularly in regions associated with catalytic activity and surface structure. Several substitutions, such as alanine at position 457 being replaced by valine (A457V) and threonine at position 448 being replaced by isoleucine (T448I), have been reported, contributing to differences in enzyme behavior. This increased variability may enhance its ability to adapt to different bacterial hosts and environmental conditions, resulting in a wider distribution and variable resistance levels [26].

mcr-4

*mcr*-4 was first described in 2017 in Italy and Spain, mainly in *Salmonella* isolates. Studies report its occurrence in both animals and humans, although its spread is still limited. It is also found in E.coli. The resistance mechanism remains the same, but gene-wise, *mcr*-4 is distinct from earlier variants and shows lower dissemination, possibly due to less efficient plasmid transfer. *mcr*-4 is considered more divergent compared to earlier variants. Structural differences are mainly observed in its transmembrane regions, which may influence how the protein is positioned within the bacterial membrane. Available data on specific mutations are limited; however, these structural variations are thought to reduce their enzymatic activity and may affect membrane localization and stability [27].

mcr-5

*mcr*-5 has been reported in environmental, animal, and some clinical samples, indicating its presence across a wide range of settings. It is most commonly associated with *Salmonella* and *E. coli*, although it has occasionally been detected in other Gram-negative bacilli. This variant was first identified in 2017 in Germany. Like other *mcr* genes, it confers resistance to colistin through modification of lipid A. *mcr*-5 shows moderate similarity to other *mcr* variants but also displays distinct characteristics. Variations near the catalytic region may influence enzyme activity, leading to differences in resistance levels. A key feature of *mcr*-5 is its strong association with mobile genetic elements, particularly transposons such as transposon number 6452 (Tn6452)-like structures. These elements enable the gene to move between different genetic locations, enhancing its persistence in diverse environments. Although specific point mutations are not commonly reported, its ability to spread through transposon-mediated transfer plays a significant role in its distribution and environmental stability [28].

mcr-6

*mcr*-6 is genetically related to *mcr*-2 and has mainly been detected in *Moraxella* species rather than common Enterobacteriaceae. Studies on this variant are limited, and it is rarely found in clinical isolates. It was reported in 2017 in the United Kingdom (UK). The mechanism is similar to other *mcr* genes, involving lipid A modification, but its restricted host range and lower mobility differentiate it from more widespread variants. *mcr*-6 shows very little difference from *mcr*-2 in terms of its amino acid composition and overall structure. Its catalytic region remains largely conserved, with only a few minor substitutions reported. These small differences do not seem to significantly affect its function, and its activity is considered similar to that of *mcr*-2. This may help explain its limited distribution and lower prevalence [29].

mcr-7

*mcr*-7 has been reported in a limited number of studies and is mainly associated with *K. pneumoniae*, particularly in clinical isolates. It was first identified in 2018 in China. The resistance mechanism remains similar to other *mcr* genes, involving lipid A modification. However, gene-level differences include their restricted spread and limited information on associated plasmid types, suggesting lower transmission potential. *mcr*-7 is related to the *mcr*-3 group and shows only minor structural differences, particularly in protein folding. Few variants have been reported so far, and these small changes are not expected to significantly alter its catalytic activity. Instead, they may influence gene expression or regulation, which could contribute to its limited distribution [30].

mcr-8

*mcr*-8 was first identified in 2018 in China and has since been increasingly reported, particularly in hospital settings. It has been detected in both human and animal isolates, most commonly in *K. pneumoniae*. Like other *mcr* genes, it confers resistance to colistin by modifying lipid A, which reduces the antibiotic’s ability to bind. However, *mcr*-8 differs from earlier variants in that it is frequently associated with MDR plasmids, increasing its clinical importance and potential for co-resistance. It is also linked with mobile genetic elements, such as insertion sequences, which facilitate its movement between bacterial populations. *mcr*-8 represents a distinct phylogenetic group among *mcr* variants. It contains stabilizing changes within its catalytic domain that may enhance enzyme function. Multiple variants have been reported, and these features are often associated with higher resistance levels. Its strong association with MDR plasmids further supports its role in the spread and persistence of multidrug resistance [31].

mcr-9

*mcr*-9 has been detected in *Salmonella*, *Enterobacter*, and *E. coli*, and several studies suggest that it is widely distributed. It was first identified in 2019 in the United States. Unlike many other *mcr* variants, it often does not show phenotypic resistance unless specific conditions induce its expression. Although it encodes a PEtN transferase that modifies lipid A, similar to other *mcr* genes, its activity is regulated, making it unique. *mcr*-9 represents a more divergent branch among *mcr* variants. It retains conserved catalytic residues required for function, but its expression is dependent on regulatory elements rather than structural changes. It typically lacks significant active mutations, and its resistance is inducible, meaning that phenotypic expression occurs only in the presence of appropriate regulatory signals. This regulatory dependence is a key feature that differentiates *mcr*-9 from other variants [32].

mcr-10

*mcr*-10 has been detected in both clinical and environmental isolates, indicating its presence across diverse settings. It was first reported in 2020 in China from Enterobacter species. Studies on this variant are still emerging, but its occurrence highlights the ongoing evolution of colistin resistance. The mechanism is similar to other *mcr* genes, involving lipid A modification; however, gene analysis shows that it is genetically distinct and associated with diverse mobile elements, suggesting potential for future spread. *mcr*-10 shares approximately 83% amino acid identity with *mcr*-9 and has a similar catalytic domain, while showing differences in its regulatory regions. Only a few variants have been reported so far. Its resistance appears to be expression-dependent, and it may represent an evolutionary intermediate between earlier and more recently identified *mcr* variants [33]. The spread of these genes is mainly driven by horizontal gene transfer through conjugative plasmids, insertion sequences, and transposons, which enable rapid movement across bacterial species and geographic regions [34]. However, the distribution of *mcr* genes is not uniform worldwide. Higher prevalence rates are reported in Asia, particularly in China, Southeast Asia, and parts of the Indian subcontinent, likely reflecting the extensive use of colistin in both clinical and agricultural settings [35]. In contrast, Europe generally shows lower prevalence, although localized increases and regional clusters have been observed, especially in Southern and Eastern regions [36]. Data from Africa and South America remain limited but are gradually increasing, with reports of multiple *mcr* variants, including *mcr*-1, *mcr*-3, *mcr*-5, *mcr*-8, and *mcr*-9, in clinical, animal, and environmental samples [37]. A comparative overview of the major characteristics of *mcr* variants from *mcr*-1 to *mcr*-10 is summarized in Table 1.

**Table 1:** Comparative overview of mcr-1 to mcr-10 Asp: aspartic acid; ColE10: Colicin E10 plasmid replicon type; Glu: glutamic acid; His: histidine; MDR: multidrug resistant; *mcr*: mobile colistin resistance; Thr: threonine; Tn: transposon number

Variant	Firstly Reported (Year, Country)	Main Bacterial Hosts	Common Reservoirs	Major Mobile Elements	Structural/Functional Characteristics	Geographic Distribution	Clinical Relevance
*mcr*-1	2015, China	*Escherichia coli*, *Klebsiella pneumoniae*	Humans, animals, food, environment	IncI2, IncX4, IncHI2 plasmids; ISApl1	Conserved catalytic domain with stable enzymatic activity; key residues include His395, Asp465, Glu246 and Thr285	Widely distributed globally (>60 countries)	Most prevalent and clinically important *mcr* variant; prototype mobile colistin resistance gene [24]
*mcr*-2	2016, Belgium	Escherichia coli	Livestock, especially pigs	Plasmids with limited dissemination	Approximately 80% amino acid identity with *mcr*-1; minor catalytic-region substitutions	Limited global distribution	Lower prevalence and transmission efficiency compared to *mcr*-1 [25]
*mcr*-3	2017, China	*Escherichia coli*, *Salmonella*,* Aeromonas*	Humans, animals, aquatic environments	Plasmids and insertion sequences	High genetic diversity with substitutions such as A457V and T448I	Predominantly Asia with sporadic global reports	Broad host range and variable resistance levels [26]
*mcr*-4	2017, Italy and Spain	*Salmonella*, *Escherichia coli*	Animals and humans	ColE10 small non-self-conjugative plasmid	Divergent transmembrane regions affecting membrane localization and stability	Mainly Europe with limited dissemination	Lower transfer efficiency and reduced spread [27]
*mcr*-5	2017, Germany	*Salmonella*, *Escherichia coli*	Environment, animals, clinical isolates	Tn6452-like transposons	Variations near catalytic region; strong transposon-associated mobility	Scattered global reports	Enhanced environmental persistence through transposon-mediated transfer [28]
*mcr*-6	2017, United Kingdom	Moraxella spp.	Animal-associated reservoirs	Limited plasmid association	Structurally very similar to *mcr*-2 with conserved catalytic region	Rare and geographically restricted	Limited clinical significance and restricted host range [29]
*mcr*-7	2018, China	Klebsiella pneumoniae	Clinical isolates	Plasmids (limited data available)	Minor structural differences related to the *mcr*-3 group	Mainly reported in Asia	Limited dissemination and low transmission potential [30]
*mcr*-8	2018, China	Klebsiella pneumoniae	Humans and animals	MDR plasmids and insertion sequences	Stabilizing catalytic-domain changes associated with enhanced enzyme activity	Increasing global reports, especially in Asia	Frequently associated with multidrug resistance and hospital-associated isolates [31]
*mcr*-9	2019, United States	*Salmonella*,* Enterobacter*,* Escherichia coli*	Humans, animals, environment	Plasmids with regulatory elements	Conserved catalytic residues but inducible expression dependent on regulatory signals	Widely distributed	Often phenotypically silent unless induced under specific conditions [32]
*mcr*-10	2020, China	Enterobacter spp.	Clinical and environmental sources	Diverse mobile genetic elements	Approximately 83% amino acid identity with *mcr*-9; expression-dependent resistance	Emerging worldwide distribution	Potential for future dissemination and evolutionary significance [33]

**Figure 1:**
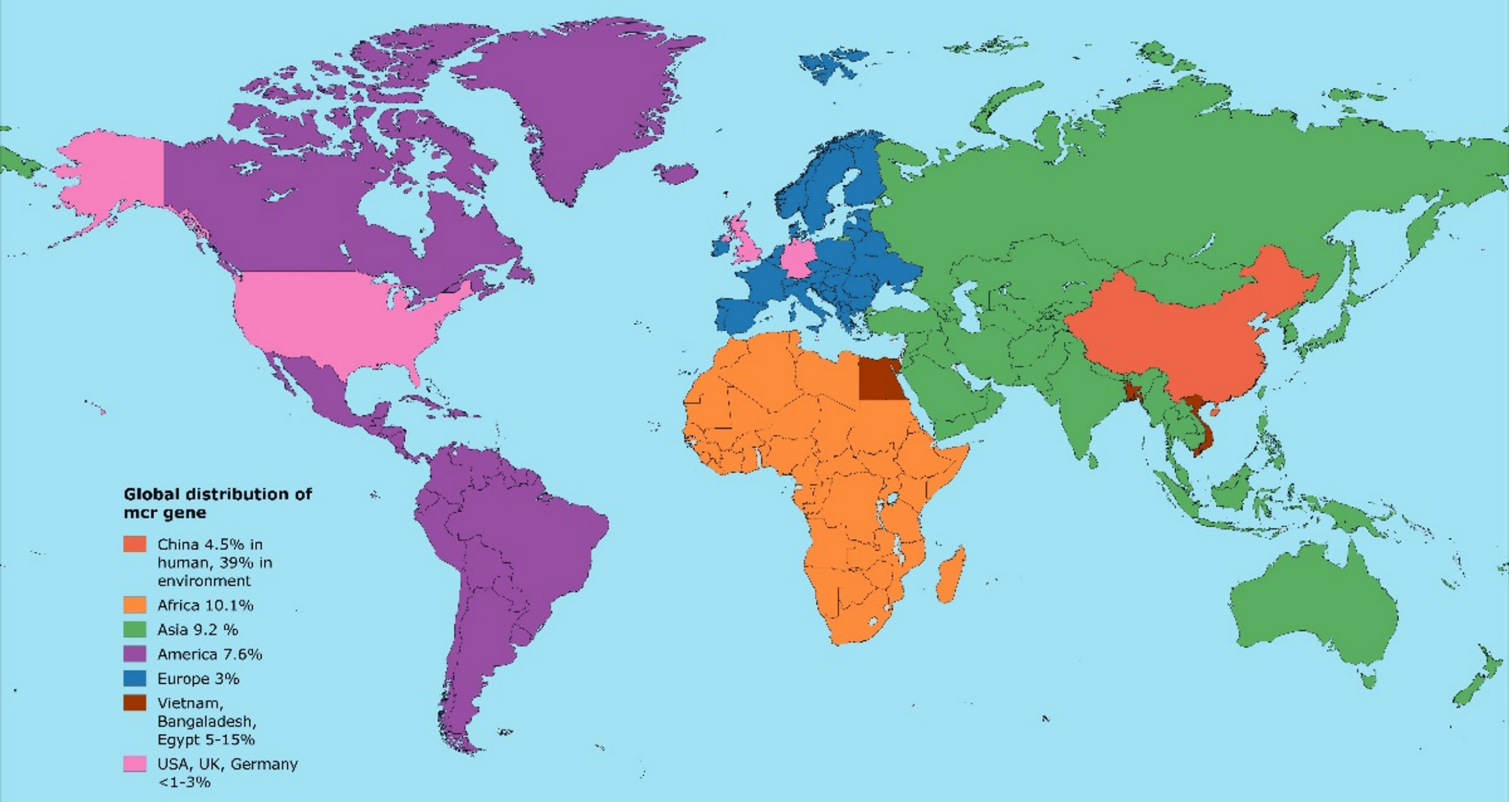
Global prevalence of mobile colistin resistance (mcr) genes *mcr: *mobile colistin resistance Image credit: The authors. Figure created with mapchart.net.

The global spread of *mcr* genes is further influenced by factors such as international travel, food trade, and environmental transmission, highlighting the importance of coordinated surveillance under a One Health approach [38]. The global prevalence and distribution of *mcr* genes are illustrated in Figure 1.

Prevalence estimates are derived from selected studies published between 2005 and 2025 included in this review. Regional percentages are based on data reported in eligible studies according to predefined inclusion criteria and should be interpreted with caution due to variability in surveillance methods. Africa (~10.1%) and Asia (~9.2%) show the highest regional prevalence, followed by the Americas (~7.6%) and Europe (~3.0%). China reports the highest country-level burden (ranging from 4.5% in human isolates to 39% in environmental samples), while Vietnam, Bangladesh, and Egypt also demonstrate elevated prevalence in animal and environmental sources. In contrast, developed countries, such as the United States of America (USA), the UK, and Germany, report comparatively lower prevalence rates (<1-3%). Overall, these estimates are descriptive and may vary due to differences in study design, surveillance systems, and diagnostic methodologies across regions. [39].

Clinical implications of colistin resistance

Therapeutic Challenges

Colistin is considered a last-line antibiotic for the treatment of infections caused by MDR and extensively drug-resistant (XDR) Gram-negative pathogens, including *P. aeruginosa* [23]. The emergence of colistin resistance severely limits available therapeutic options, often leaving clinicians with few or no effective antibiotics [9]. Treatment of colistin-resistant Gram-negative bacilli, including *P. aeruginosa* infections, is further complicated by the lack of standardized susceptibility testing, the phenomenon of heteroresistance, and the narrow therapeutic window of colistin due to nephrotoxicity and neurotoxicity [34]. Combination therapies (e.g., colistin with carbapenems, aminoglycosides, or rifampicin) are frequently employed [40]. However, clinical evidence supporting their efficacy against colistin-resistant isolates remains inconsistent [41].

Impact on Patient Outcomes

Infections caused by colistin-resistant Gram-negative bacilli are associated with delayed initiation of effective therapy, prolonged hospital stays, and increased healthcare costs [42]. Resistance often emerges during therapy, particularly in critically ill patients receiving prolonged or suboptimal dosing, leading to treatment failure and recurrent infections [43]. Colistin resistance is especially problematic in intensive care unit (ICU) settings, where patients are immunocompromised, mechanically ventilated, or have invasive devices, all of which increase susceptibility to severe Gram-negative bacilli bacterial infections [44]. Studies consistently demonstrate poorer clinical outcomes in patients infected with colistin-resistant strains compared with colistin-susceptible counterparts [45]. Multiple clinical studies have reported that colistin resistance is independently associated with increased mortality and morbidity, particularly in bloodstream infections, ventilator-associated pneumonia, and septic shock [46]. Mortality rates in patients infected with colistin-resistant Gram-negative bacilli have been reported to exceed 40-60%, depending on infection site and patient comorbidities [47]. In *P. aeruginosa*, colistin resistance often coexists with resistance to other antibiotic classes, resulting in pan-drug-resistant (PDR) phenotypes, which are strongly linked to poor prognosis and limited survival [45]. From a public health perspective, the spread of plasmid-mediated colistin resistance genes (e.g., *mcr*) further amplifies the clinical burden by enabling rapid dissemination of resistance across species and healthcare settings [2].

Detection and diagnostic considerations

Phenotypic Methods

Phenotypic detection of colistin resistance remains challenging due to the poor diffusion of polymyxins in agar, heteroresistance, and variability among testing platforms. Consequently, routine disk diffusion and gradient diffusion methods (e.g., Epsilometer test) are not recommended for colistin susceptibility testing. Broth microdilution (BMD) is considered the gold standard method for determining colistin minimum inhibitory concentrations (MICs) in Gram-negative bacilli, including *P. aeruginosa* [48]. International guidelines from the European Committee on Antimicrobial Susceptibility Testing (EUCAST) and the Clinical and Laboratory Standards Institute (CLSI) endorse BMD using cation-adjusted Mueller-Hinton broth without surfactants to ensure reliable results [49]. Several commercial BMD-based assays have been developed to simplify routine testing, showing variable but generally acceptable performance compared with reference BMD [50]. Rapid phenotypic assays, to detect colistin resistance based on glucose metabolism in the presence of colistin and provide results within two to four hours; however, their performance in *P. aeruginosa* is less consistent than in Enterobacterales [51].

Genotypic Methods

Genotypic methods enable the detection of known genetic determinants of colistin resistance, particularly plasmid-mediated *mcr* genes, and are essential for surveillance and infection control [52]. Polymerase chain reaction (PCR) assays targeting *mcr*-1 to *mcr*-10 are widely used as genotypic detection [18]. Whole-genome sequencing (WGS) provides a comprehensive approach for identifying chromosomal mutations associated with colistin resistance, such as alterations in *pmrAB*, *phoPQ*, *parRS*, *colRS*, and *cprRS*, as well as the presence and genetic context of *mcr* genes [53]. WGS is increasingly used in reference laboratories to support outbreak investigations and track the global dissemination of resistance determinants [54].

Epidemiology and prevalence of colistin resistance in clinical isolates

Surveillance Reports

Global surveillance programs have consistently reported an increasing prevalence of colistin resistance among clinically important Gram-negative pathogens, particularly *P. aeruginosa*, *Acinetobacter baumannii*, and carbapenem-resistant Enterobacterales (CRE) [55]. Resistance has been most frequently documented in ICUs, where selective pressure from last-line antibiotic use is high [44]. Large international surveillance initiatives, such as the SENTRY Antimicrobial Surveillance Program, the European Antimicrobial Resistance Surveillance Network (EARS-Net), and national antimicrobial resistance monitoring programs, indicate that colistin resistance in *P. aeruginosa* has historically remained relatively low, generally reported at <5% during the early 2000s. However, more recent data from the last decade (approximately 2010-2020) show a gradual increase, with resistance rates rising to around 5-10% in certain regions, particularly among MDR and XDR isolates [56]. In comparison, resistance rates in *A. baumannii* are consistently higher. Importantly, colistin resistance in *P. aeruginosa* is frequently reported to emerge during the course of therapy, highlighting its strong adaptive capacity under antimicrobial pressure [57].

Prevalence of mcr Genes in Clinical Isolates

The prevalence of plasmid-mediated *mcr* genes in clinical isolates varies widely depending on bacterial species, geographic region, and surveillance methodology [18]. Overall, *mcr* genes are most prevalent in Enterobacterales, particularly *E. coli* and *K. pneumoniae*, while their occurrence in *P. aeruginosa* remains relatively rare [20]. Clinical studies have reported *mcr* gene prevalence ranging from <1% to >20% in Enterobacterales isolates in some high-burden regions [58]. In Gram-negative organisms, multiple *mcr* gene variants, ranging from *mcr*-1 to *mcr*-10, have been identified, with *mcr*-1 being the most widely reported globally. These genes are commonly detected in species such as *E. coli *and *K. pneumoniae*, whereas *mcr*-positive *P. aeruginosa* isolates are reported only sporadically and at very low frequencies (typically <1-2%). However, occasional cases and localized outbreaks involving *mcr*-1 and *mcr*-3 have been documented, indicating the potential for further spread [22]. Despite the low prevalence, the detection of *mcr* genes in *P. aeruginosa *is clinically significant due to the organism’s intrinsic resistance and its capacity to acquire multiple resistance mechanisms [59]. Reports of *mcr*-positive *P. aeruginosa* remain sporadic, with limited evidence supporting sustained outbreak transmission, as most resistance in this organism is driven by chromosomal and intrinsic mechanisms rather than plasmid-mediated spread [20,60].

Infection control and stewardship

Antibiotic Stewardship

Antibiotic stewardship programs play a critical role in limiting the emergence and spread of colistin resistance by promoting the judicious use of last-line antimicrobials [61]. Given the narrow therapeutic index and toxicity profile of colistin, stewardship strategies emphasize its restricted use, optimized dosing based on pharmacokinetic/pharmacodynamic (PK/PD) principles, and avoidance of inappropriate empirical therapy [40]. Implementation of Antimicrobial Stewardship Programs has been associated with reduced colistin consumption, decreased selective pressure, and lower rates of MDR Gram-negative infections, including *P. aeruginosa* [62]. In addition, stewardship interventions such as antibiotic de-escalation, combination therapy optimization, and treatment duration control have been shown to improve clinical outcomes while minimizing resistance development [63].

Surveillance

Hospital-based surveillance programs, supported by national and international networks, such as Global Antimicrobial Resistance and Use Surveillance System (GLASS), EARS-Net, and SENTRY Antimicrobial Surveillance Program, provide critical data on resistance trends and inform infection control policies [55]. Integration of human, animal, and environmental surveillance within a One Health framework is increasingly recognized as essential to control the global spread of colistin resistance [64].

Hygiene Measures

Strict infection prevention and control (IPC) measures are fundamental to reducing the transmission of colistin-resistant organisms in healthcare settings [65]. Core strategies include hand hygiene compliance, use of personal protective equipment (PPE), contact precautions, and environmental cleaning and disinfection [66]. In high-risk units such as ICUs, additional measures such as active surveillance cultures, patient cohorting, and isolation of colonized or infected patients have been shown to limit nosocomial transmission of MDR and XDR *P. aeruginosa* [67]. Education and continuous training of healthcare workers are critical to ensure sustained adherence to hygiene protocols and stewardship policies [68]. 

Future perspectives

The increasing emergence of colistin resistance in *P. aeruginosa* highlights the urgent need for new therapeutic strategies to manage MDR and XDR Gram-negative infections [69]. New-generation polymyxin derivatives retain strong antibacterial activity while exhibiting reduced nephrotoxicity and represent promising alternatives to conventional colistin therapy [60]. Combination regimens involving colistin or its derivatives with beta-lactams may further enhance antimicrobial efficacy and help overcome resistance [70]. From a One Health perspective, environmental and ecological studies are essential to understand the reservoirs and transmission pathways of colistin resistance [64]. The widespread detection of *mcr* genes in wastewater, livestock, wildlife, food products, and natural ecosystems highlights the role of environmental dissemination in resistance spread. Advances in WGS and genomic epidemiology have improved the detection of colistin resistance determinants and the tracking of high-risk clones and resistance plasmids, supporting more effective surveillance and antimicrobial stewardship strategies [71].

Limitation

This review has certain limitations. Limited data from underrepresented regions, particularly Africa and South America, may affect the global generalizability of the findings and contribute to regional data gaps in prevalence estimates. Additionally, heterogeneity in study design, sample size, detection methods, and surveillance systems across studies may influence the comparability of reported results. As this review is based on a narrative synthesis approach, no quantitative meta-analysis or statistical pooling was performed, which may limit direct comparison across studies.

## Conclusions

Colistin resistance in Gram-negative bacilli represents a growing global threat, driven increasingly by plasmid-mediated *mcr* genes. Although *mcr*-mediated resistance remains relatively rare in *P. aeruginosa* compared to Enterobacterales, its potential for horizontal transfer and coexistence with other multidrug-resistance determinants poses serious therapeutic and infection control challenges. Early detection through phenotypic and genotypic surveillance, combined with antibiotic stewardship and strict infection control measures, is essential to limit dissemination. The research should focus on developing new treatment options, studying the genetic spread of resistance, and understanding how resistant strains affect the environment, so that better strategies can be created and the effectiveness of last-line antibiotics can be maintained. These findings should be interpreted in the context of methodological variability across studies.

## References

[1] (2026). World Health Organization. Global Antimicrobial Resistance and Use Surveillance System (GLASS) Report 2024-2025. https://www.who.int/initiatives/glass.

[2] Falagas ME, Kasiakou SK (2005). Colistin: the revival of polymyxins for the management of multidrug-resistant gram-negative bacterial infections. Clin Infect Dis.

[3] Moradali MF, Ghods S, Rehm BH (2017). Pseudomonas aeruginosa lifestyle: a paradigm for adaptation, survival and persistence. Front Cell Infect Microbiol.

[4] Bassetti M, Vena A, Croxatto A, Righi E, Guery B (2018). How to manage Pseudomonas aeruginosa infections. Drugs Context.

[5] Poirel L, Jayol A, Nordmann P (2017). Polymyxins: antibacterial activity, susceptibility testing, and resistance mechanisms encoded by plasmids or chromosomes. Clin Microbiol Rev.

[6] Liu YY, Wang Y, Walsh TR (2016). Emergence of plasmid-mediated colistin resistance mechanism MCR-1 in animals and human beings in China: a microbiological and molecular biological study. Lancet Infect Dis.

[7] Partridge SR, Kwong SM, Firth N, Jensen SO (2018). Mobile genetic elements associated with antimicrobial resistance. Clin Microbiol Rev.

[8] Sharma S, Devkota MD, Pokhrel BM, Banjara MR (2023). Detection of bla(NDM-1,)mcr-1 and MexB in multidrug resistant Pseudomonas aeruginosa isolated from clinical specimens in a tertiary care hospital of Nepal. BMC Microbiol.

[9] El-Sayed Ahmed MA, Zhong LL, Shen C, Yang Y, Doi Y, Tian GB (2020). Colistin and its role in the era of antibiotic resistance: an extended review (2000-2019). Emerg Microbes Infect.

[10] Moskowitz SM, Ernst RK, Miller SI (2004). PmrAB, a two-component regulatory system of Pseudomonas aeruginosa that modulates resistance to cationic antimicrobial peptides and addition of aminoarabinose to lipid A. J Bacteriol.

[11] Lee JY, Ko KS (2014). Mutations and expression of PmrAB and PhoPQ related with colistin resistance in Pseudomonas aeruginosa clinical isolates. Diagn Microbiol Infect Dis.

[12] Jeannot K, Bolard A, Plésiat P (2017). Resistance to polymyxins in Gram-negative organisms. Int J Antimicrob Agents.

[13] Fernández L, Hancock RE (2012). Adaptive and mutational resistance: role of porins and efflux pumps in drug resistance. Clin Microbiol Rev.

[14] Li XZ, Plésiat P, Nikaido H (2015). The challenge of efflux-mediated antibiotic resistance in Gram-negative bacteria. Clin Microbiol Rev.

[15] Skov RL, Monnet DL (2016). Plasmid-mediated colistin resistance (mcr-1 gene): three months later, the story unfolds. Euro Surveill.

[16] Mulcahy LR, Isabella VM, Lewis K (2014). Pseudomonas aeruginosa biofilms in disease. Microb Ecol.

[17] Olaitan AO, Morand S, Rolain JM (2014). Mechanisms of polymyxin resistance: acquired and intrinsic resistance in bacteria. Front Microbiol.

[18] Wang C, Feng Y, Liu L, Wei L, Kang M, Zong Z (2020). Identification of novel mobile colistin resistance gene mcr-10. Emerg Microbes Infect.

[19] Sun J, Zhang H, Liu YH, Feng Y (2018). Towards understanding MCR-like colistin resistance. Trends Microbiol.

[20] Khuntayaporn P, Thirapanmethee K, Chomnawang MT (2022). An update of mobile colistin resistance in non-fermentative gram-negative bacilli. Front Cell Infect Microbiol.

[21] Hameed F, Khan MA, Muhammad H, Sarwar T, Bilal H, Rehman TU (2019). Plasmid-mediated mcr-1 gene in Acinetobacter baumannii and Pseudomonas aeruginosa: first report from Pakistan. Rev Soc Bras Med Trop.

[22] Davoodi NR, Soleimani N, Hosseini SM, Rahnamaye-Farzami M (2025). Co-occurrence of mcr-1 and mcr-3 mobilized colistin resistance genes among carbapenem-resistant Pseudomonas aeruginosa in Iran. Sci Rep.

[23] Li J, Nation RL, Turnidge JD, Milne RW, Coulthard K, Rayner CR, Paterson DL (2006). Colistin: the re-emerging antibiotic for multidrug-resistant Gram-negative bacterial infections. Lancet Infect Dis.

[24] Matamoros S, van Hattem JM, Arcilla MS (2017). Global phylogenetic analysis of Escherichia coli and plasmids carrying the mcr-1 gene indicates bacterial diversity but plasmid restriction. Sci Rep.

[25] Xavier BB, Lammens C, Ruhal R, Kumar-Singh S, Butaye P, Goossens H, Malhotra-Kumar S (2016). Identification of a novel plasmid-mediated colistin-resistance gene, mcr-2, in Escherichia coli, Belgium, June 2016. Euro Surveill.

[26] Yin W, Li H, Shen Y (2017). Novel plasmid-mediated colistin resistance gene mcr-3 in Escherichia coli. mBio.

[27] Carattoli A, Villa L, Feudi C (2017). Novel plasmid-mediated colistin resistance mcr-4 gene in Salmonella and Escherichia coli, Italy 2013, Spain and Belgium, 2015 to 2016. Euro Surveill.

[28] Borowiak M, Fischer J, Hammerl JA, Hendriksen RS, Szabo I, Malorny B (2017). Identification of a novel transposon-associated phosphoethanolamine transferase gene, mcr-5, conferring colistin resistance in d-tartrate fermenting Salmonella enterica subsp. enterica serovar Paratyphi B. J Antimicrob Chemother.

[29] AbuOun M, Stubberfield EJ, Duggett NA (2017). mcr-1 and mcr-2 variant genes identified in Moraxella species isolated from pigs in Great Britain from 2014 to 2015. J Antimicrob Chemother.

[30] Yang YQ, Li YX, Lei CW, Zhang AY, Wang HN (2018). Novel plasmid-mediated colistin resistance gene mcr-7.1 in Klebsiella pneumoniae. J Antimicrob Chemother.

[31] Wang X, Wang Y, Zhou Y (2018). Emergence of a novel mobile colistin resistance gene, mcr-8, in NDM-producing Klebsiella pneumoniae. Emerg Microbes Infect.

[32] Carroll LM, Gaballa A, Guldimann C, Sullivan G, Henderson LO, Wiedmann M (2019). Identification of novel mobilized colistin resistance gene mcr-9 in a multidrug-resistant, colistin-susceptible Salmonella enterica serotype typhimurium isolate. mBio.

[33] Xu T, Zhang C, Ji Y, Song J, Liu Y, Guo Y, Zhou K (2021). Identiﬁcation of mcr-10 carried by self-transmissible plasmids and chromosome in Enterobacter roggenkampii strains isolated from hospital sewage water. Environ Pollut.

[34] Nation RL, Li J (2009). Colistin in the 21st century. Curr Opin Infect Dis.

[35] Petrosillo N, Taglietti F, Granata G (2019). Treatment options for colistin resistant Klebsiella pneumoniae: present and future. J Clin Med.

[36] Mmatli M, Mbelle NM, Osei Sekyere J (2022). Global epidemiology, genetic environment, risk factors and therapeutic prospects of mcr genes: a current and emerging update. Front Cell Infect Microbiol.

[37] Walsh TR, Wu Y (2016). China bans colistin as a feed additive for animals. Lancet Infect Dis.

[38] Shen Z, Wang Y, Shen Y, Shen J, Wu C (2016). Early emergence of mcr-1 in Escherichia coli from food-producing animals. Lancet Infect Dis.

[39] Wang Y, Xu C, Zhang R (2020). Changes in colistin resistance and mcr-1 abundance in Escherichia coli of animal and human origins following the ban of colistin-positive additives in China: an epidemiological comparative study. Lancet Infect Dis.

[40] Tsuji BT, Pogue JM, Zavascki AP (2019). International consensus guidelines for the optimal use of the polymyxins: endorsed by the American College of Clinical Pharmacy (ACCP), European Society of Clinical Microbiology and Infectious Diseases (ESCMID), Infectious Diseases Society of America (IDSA), International Society for Anti-infective Pharmacology (ISAP), Society of Critical Care Medicine (SCCM), and Society of Infectious Diseases Pharmacists (SIDP). Pharmacotherapy.

[41] Zusman O, Altunin S, Koppel F, Dishon Benattar Y, Gedik H, Paul M (2017). Polymyxin monotherapy or in combination against carbapenem-resistant bacteria: systematic review and meta-analysis. J Antimicrob Chemother.

[42] Kontopidou F, Plachouras D, Papadomichelakis E (2011). Colonization and infection by colistin-resistant Gram-negative bacteria in a cohort of critically ill patients. Clin Microbiol Infect.

[43] Bergen PJ, Landersdorfer CB, Lee HJ, Li J, Nation RL (2012). 'Old' antibiotics for emerging multidrug-resistant bacteria. Curr Opin Infect Dis.

[44] Vincent JL, Rello J, Marshall J (2009). International study of the prevalence and outcomes of infection in intensive care units. JAMA.

[45] Zavascki AP, Goldani LZ, Li J, Nation RL (2007). Polymyxin B for the treatment of multidrug-resistant pathogens: a critical review. J Antimicrob Chemother.

[46] Capone A, Giannella M, Fortini D (2013). High rate of colistin resistance among patients with carbapenem-resistant Klebsiella pneumoniae infection accounts for an excess of mortality. Clin Microbiol Infect.

[47] Durante-Mangoni E, Andini R, Zampino R (2019). Management of carbapenem-resistant Enterobacteriaceae infections. Clin Microbiol Infect.

[48] Chantratita N, Wikraiphat C, Tandhavanant S (2016). Comparison of community-onset Staphylococcus argenteus and Staphylococcus aureus sepsis in Thailand: a prospective multicentre observational study. Clin Microbiol Infect.

[49] Jayol A, Nordmann P, André C, Poirel L, Dubois V (2018). Evaluation of three broth microdilution systems to determine colistin susceptibility of Gram-negative bacilli. J Antimicrob Chemother.

[50] Nordmann P, Jayol A, Poirel L (2016). Rapid detection of polymyxin resistance in Enterobacteriaceae. Emerg Infect Dis.

[51] Sadek M, Tinguely C, Poirel L, Nordmann P (2020). Rapid Polymyxin/Pseudomonas NP test for rapid detection of polymyxin susceptibility/resistance in Pseudomonas aeruginosa. Eur J Clin Microbiol Infect Dis.

[52] Rebelo AR, Bortolaia V, Kjeldgaard JS (2018). Multiplex PCR for detection of plasmid-mediated colistin resistance determinants, mcr-1, mcr-2, mcr-3, mcr-4 and mcr-5 for surveillance purposes. Euro Surveill.

[53] Li J, Nation RL (2006). Old polymyxins are back: is resistance close?. Clin Infect Dis.

[54] Kieffer N, Nordmann P, Poirel L (2017). Moraxella species as potential sources of MCR-like polymyxin resistance determinants. Antimicrob Agents Chemother.

[55] (2026). World Health Organization. Global Antimicrobial Resistance and Use Surveillance System (‎GLASS)‎ report: antibiotic use data for 2022. https://www.who.int/publications/i/item/9789240108127.

[56] Jacobsson S, Cole MJ, Schröder D, Jansen van Rensburg M, Day M, Ködmön C, Unemo M (2025). Antimicrobial resistance in Neisseria gonorrhoeae and its risk groups in 23 European countries in 2022 within the European Gonococcal Antimicrobial Surveillance Programme (Euro-GASP): a retrospective observational study. Lancet Reg Health Eur.

[57] Gales AC, Jones RN, Sader HS (2011). Contemporary activity of colistin and polymyxin B against a worldwide collection of Gram-negative pathogens: results from the SENTRY Antimicrobial Surveillance Program (2006-09). J Antimicrob Chemother.

[58] Ling Z, Yin W, Shen Z, Wang Y, Shen J, Walsh TR (2020). Epidemiology of mobile colistin resistance genes mcr-1 to mcr-9. J Antimicrob Chemother.

[59] Anyanwu MU, Jaja IF, Okpala CO, Njoga EO, Okafor NA, Oguttu JW (2023). Mobile colistin resistance (mcr) gene-containing organisms in poultry sector in low- and middle-income countries: epidemiology, characteristics, and One Health control strategies. Antibiotics (Basel).

[60] Nang SC, Azad MA, Velkov T, Zhou QT, Li J (2021). Rescuing the last-line polymyxins: achievements and challenges. Pharmacol Rev.

[61] Barlam TF, Cosgrove SE, Abbo LM (2016). Implementing an antibiotic stewardship program: guidelines by the Infectious Diseases Society of America and the Society for Healthcare Epidemiology of America. Clin Infect Dis.

[62] Rice LB (2018). Antimicrobial stewardship and antimicrobial resistance. Med Clin North Am.

[63] Paul M, Daikos GL, Durante-Mangoni E (2018). Colistin alone versus colistin plus meropenem for treatment of severe infections caused by carbapenem-resistant Gram-negative bacteria: an open-label, randomised controlled trial. Lancet Infect Dis.

[64] Robinson TP, Bu DP, Carrique-Mas J (2016). Antibiotic resistance is the quintessential One Health issue. Trans R Soc Trop Med Hyg.

[65] Siegel JD, Rhinehart E, Jackson M, Chiarello L (2007). Management of multidrug-resistant organisms in health care settings, 2006. Am J Infect Control.

[66] Pittet D, Allegranzi B, Boyce J (2009). The World Health Organization guidelines on hand hygiene in health care and their consensus recommendations. Infect Control Hosp Epidemiol.

[67] Santos RP, Mayo TW, Siegel JD (2008). Healthcare epidemiology: active surveillance cultures and contact precautions for control of multidrug-resistant organisms: ethical considerations. Clin Infect Dis.

[68] Erasmus V, Daha TJ, Brug H, Richardus JH, Behrendt MD, Vos MC, van Beeck EF (2010). Systematic review of studies on compliance with hand hygiene guidelines in hospital care. Infect Control Hosp Epidemiol.

[69] Bassetti M, Peghin M, Vena A, Giacobbe DR (2019). Treatment of infections due to MDR Gram-negative bacteria. Front Med (Lausanne).

[70] Mahlapuu M, Håkansson J, Ringstad L, Björn C (2016). Antimicrobial peptides: an emerging category of therapeutic agents. Front Cell Infect Microbiol.

[71] Ellington MJ, Ekelund O, Aarestrup FM (2017). The role of whole genome sequencing in antimicrobial susceptibility testing of bacteria: report from the EUCAST Subcommittee. Clin Microbiol Infect.

